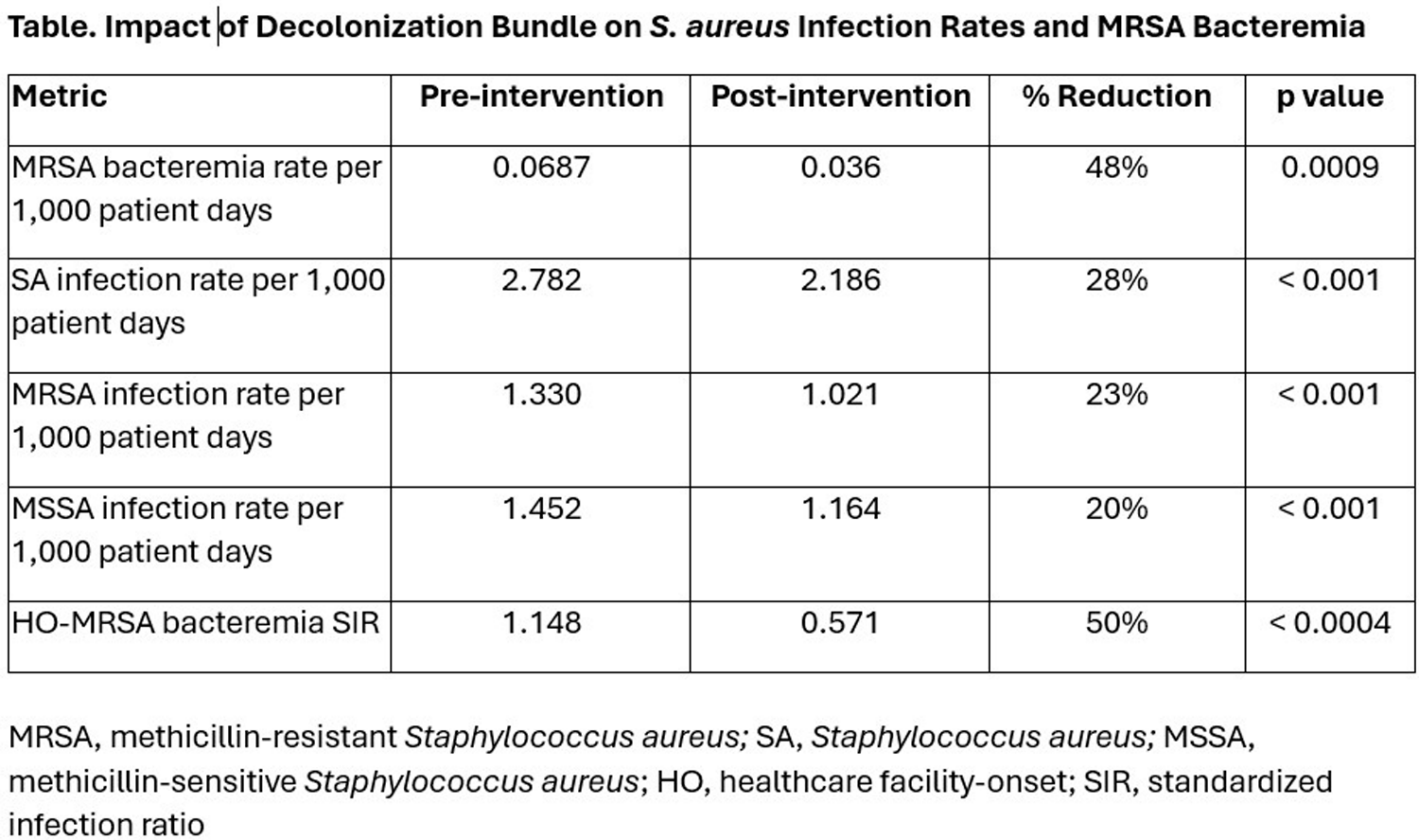# 190 Large Drug-resistant Klebsiella pneumoniae Outbreak in an Acute Care Hospital — Indiana, United States, 2025

**DOI:** 10.1017/ash.2026.10440

**Published:** 2026-06-23

**Authors:** Geehan Suleyman, Eman Chami, Kim Semelroth, Hannah Musgrove, Abigail Ruby, Anita Shallal

**Affiliations:** 1 Henry Ford Health; 2 Henry Ford Health System; 3 Henry Ford Hospital

## Abstract

**Background:** Staphylococcus aureus (SA) is a leading cause of infection-related mortality worldwide and remains one of the most common pathogens in healthcare settings, particularly methicillin-resistant SA (MRSA). Approximately 30% of patients are colonized with SA in the nares, and these asymptomatic carriers serve as a reservoir for invasive infections, including bloodstream infections, pneumonia, and surgical site infections. Targeted strategies, such as nasal decolonization, are essential to reduce colonization and prevent subsequent infection. We evaluated the impact of SA nasal decolonization on overall SA infection rates and healthcare facility-onset (HO) MRSA bacteremia, a laboratory-identified (LabID) event. **Methods:** In this pre–post quasi-experimental retrospective study, we compared overall SA, MRSA, and methicillin-susceptible SA (MSSA) infection rates per 1,000 patient days and the HO-MRSA bacteremia Standardized Infection Ratio (SIR) between the preintervention period (January 2020–June 2021) and the intervention period (October 2023–September 2025) at a 5-hospital healthcare system in Michigan. HO-MRSA bacteremia was determined using National Healthcare Safety Network (NHSN) criteria. SA infections included positive clinical cultures in hospitalized patients. Previously, our protocol consisted of daily chlorhexidine (CHG) bathing only, and HO-MRSA bacteremia SIR remained above goal. In a two-phased approach, we implemented nasal decolonization with an alcohol-based nasal antiseptic twice daily in addition to CHG for all ICU patients in August 2021, followed by all general practice unit patients with central lines in August 2023. **Results:** Following implementation, the cumulative HO-MRSA bacteremia SIR decreased 50% from 1.148 to 0.571 (95% CI, 0.336–0.73; p. **Conclusion:** Adding nasal decolonization to daily CHG bathing significantly reduced SA infection rates and HO-MRSA bacteremia. These findings confirm the effectiveness of a bundled decolonization strategy in mitigating both MRSA and MSSA infections. Broader adoption of nasal antiseptic protocols, alongside CHG bathing, should be considered a critical component of infection prevention strategy for high-risk hospital populations.

**Figure:**